# Role of Sulfur Compounds in Vegetable and Mushroom Aroma

**DOI:** 10.3390/molecules27186116

**Published:** 2022-09-19

**Authors:** Monika A. Marcinkowska, Henryk H. Jeleń

**Affiliations:** Faculty of Food Science and Nutrition, Poznań University of Life Sciences, Wojska Polskiego 31, 60-624 Poznań, Poland

**Keywords:** sulfur compounds, vegetable aroma, glucosinolates, isothiocyanates, nitriles, sulfides, polysulfides, thiols, *Brassica* vegetables

## Abstract

At the base of the food pyramid is vegetables, which should be consumed most often of all food products, especially in raw and unprocessed form. Vegetables and mushrooms are rich sources of bioactive compounds that can fulfill various functions in plants, starting from protection against herbivores and being natural insecticides to pro-health functions in human nutrition. Many of these compounds contain sulfur in their structure. From the point of view of food producers, it is extremely important to know that some of them have flavor properties. Volatile sulfur compounds are often potent odorants, and in many vegetables, belonging mainly to *Brassicaeae* and Allium (*Amaryllidaceae*), sulfur compounds determine their specific flavor. Interestingly, some of the pathways that form volatile sulfur compounds in vegetables are also found in selected edible mushrooms. The most important odor-active organosulfur compounds can be divided into isothiocyanates, nitriles, epithionitriles, thiols, sulfides, and polysulfides, as well as others, such as sulfur containing carbonyl compounds and esters, R-L-cysteine sulfoxides, and finally heterocyclic sulfur compounds found in shiitake mushrooms or truffles. This review paper summarizes their precursors and biosynthesis, as well as their sensory properties and changes in selected technological processes.

## 1. Introduction

The intake of vegetables plays a crucial role in maintaining the homeostasis of the human body due to bioactive compounds that are present in plant tissues. Sulfur is a fundamental element, ensuring the sustainable development of plants and people. Amino acids, such as methionine and cysteine, derived originally from plants, represent the main source of sulfur for the human diet [1]. Vegetables contain vitamins, polyphenols, fiber, and micronutrients, which are necessary for daily diets, and they can contribute to the prevention of chronic diseases [2,3]. Sulfur-containing compounds are commonly present in Brassica vegetables, namely broccoli, cabbage, and cauliflower, or Allium vegetables, for example, garlic, leeks, and onions. The structure of organosulfur compounds is based on sulfur atoms that are bound with a cyanate group or a carbon atom in a chain or cyclic arrangement. The bioactive compounds present in Allium species are alk(en)yl cysteine sulfoxides; S-allyl cysteine; thiosulfinates; diallyl; mono-, di-, and tri-sulfides; and vinyldithiins, while for cruciferous vegetables the largest group are glucosinolates (GLSs) and their breakdown produces isothiocyanates (ITCs) [4]. An equally important role of GLSs in plants is their self-protection strategies. The first mechanism of food protection involves repelling pests by releasing ITCs, whereas the attraction of the natural enemies of insects is linked with the effects of nitriles [5]. Depending on the condition of GLSs degradation, such as the company of additional cofactors and proteins, presence of metal ions, or pH value, it is possible to obtain other rearranged products, namely epithionitriles, nitriles, oxazolidine-2-thiones, and thiocyanates [6]. Volatile sulfur compounds contribute significantly to the characteristic aroma of vegetables belonging to discussed above Brassica and Allium, being usually among their key odorants. It makes them a very unique group of plant metabolites, which are interesting from a flavor chemistry and also analytical point of view due to their diversity, low odor thresholds, instability, and reactivity.

Although mushroom-like flavor is usually associated with eight carbon unsaturated alcohols and ketones (mainly 1-octene-3-ol, 3-octanone, 1-octene-3-one), sulfur compounds are also important components of some mushrooms, particularly shiitake and truffles, which are discussed in this review. Due to their high price, truffles are used as food seasoning rather than vegetables, unlike shiitake. Heterocyclic organosulfur compounds provide burned, sulfuric, onion-like, rubber-like, and truffle-like odor notes [7,8]. Although mushrooms are consumed mainly for their sensory features, especially aroma, they also are valuable sources of valuable bioactive compounds. Shiitake mushrooms are a low energy-dense food and supply nutritionally relevant amounts of B group vitamins and minerals; they may be a proper source of vitamin D_2_, and their consumption has some medicinally beneficial properties, such as improved immunity [9]. Truffles are valuable sources of dietary fiber, essential amino acids, metals, and ergosteroids and have many health-promoting properties, particularly antioxidant, anti-inflammatory, antimicrobial property, immunomodulatory, antitumor properties, and anti-depressant properties [10].

Sulfur compounds as metabolites show various chemical structures due to their differences in molecular weight, stability, polarity, and volatility. They are present in food and may be formed or altered as a result of enzymatic and chemical reactions in technological processes and during storage. As a result of heating, smoking, cooking, frying, or roasting, many complex reactions, namely oxidation, degradation, hydrolysis, dehydration, condensation, and decarboxylation, may take place. Volatile sulfur compounds (VSCs) in food can be divided into several classes according to their functional groups (Table 1) [11]. Thiols (mercaptans) constitute a wide group with antioxidant properties that are labeled as part of the sulfhydryl functional group [12]. Methanethiol represents the parent compounds, as a result of easy oxidation into sulfides (dimethyl sulfide, dimethyl disulfide, and dimethyl trisulfide) [13]. Further oxidation of sulfides can lead to sulfoxides or sulfones. The variety of sulfur compounds is connected to the ability of sulfur atoms for taking different oxidation states, ranging from −2 in sulfides, 0 in sulfoxides, and +2 in sulfones [14]. In addition to the sulfur compounds resulting from the breakdown of GLSs mentioned above, an important group is S-heterocyclic compounds. Thiophenes, with a smoky, sulfurous, and roasted aroma, are created during thermal processes, especially when bioavailability of sulfur is sufficient by a high concentration of cysteine [15]. Thiazolines and thiazoles are formed under thermal processes in the Maillard reaction. Thiazoles are associated with a roasted aroma in food [16]. The 2-acetyl-2-thiazoline is a crucial flavor compound with a lower detection threshold at 1 ppm level, with a pleasant popcorn-like, roasted, nutty aroma [17].

**Figure 1:**
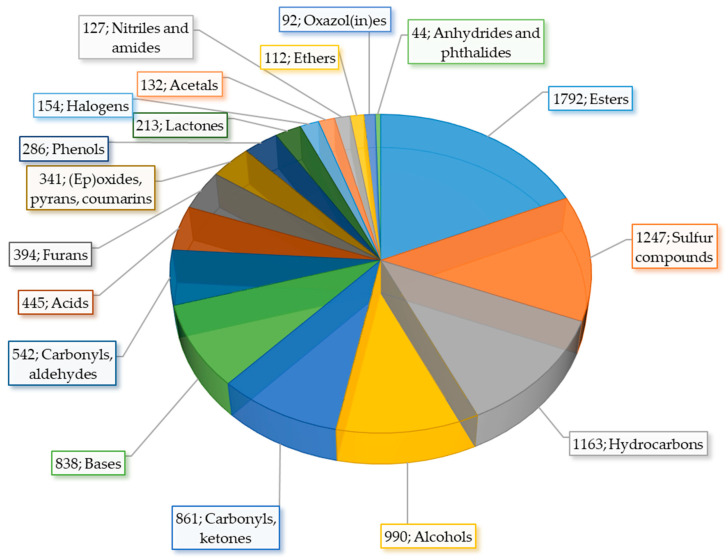
Volatile compounds in food according to volatile compounds in food database based on [28].

Sulfur compounds present in such complex matrices as food are an analytical challenge due to their often sub-parts-per-billion concentrations. As a result of low sensory detection thresholds, VSCs are emphatically responsible for the aroma creation of food. Diallyl disulfide (DADS) is an aroma impact compound for garlic and allyl isothiocyanate causes a typical mustard flavor. However, not all sulfur compounds are a backbone of vegetable aroma; some of them are responsible for sensory nuances in total flavor. Moreover, flavor sensory impact relies on specific odor threshold and level of content in particular food [27]. Nevertheless, sulfur compounds have a significant impact in creating food aroma also because they are the second largest category in volatile constituents in food after esters (Figure 1). Sulfur compounds can be grouped into many distinct categories, which include ITCs, nitriles, epithethriles, sulfides, polysulfides, and thiols, as well as a wide group of miscellaneous compounds.

The main aim of this study is to summarize information and highlight the basic function of sulfur compounds in the creation of the aroma of vegetables and mushrooms. Although the main focus is on vegetables as a source of sulfur aroma compounds (mainly Allium and Brassica), mushrooms were included in the review, as some of their volatiles characteristics are shared with Allium vegetables, partially the pathways in the formation of aroma compounds. The formation of sulfur aroma compounds and their precursors are summarized, as well as their role as key odorants. The influence of food processing, namely cooking, baking, freezing, and frying, on sulfur compounds will be discussed. Apart from their aroma, sulfur compounds are also crucial in the creation of the taste of vegetables, especially Brassica; however, the present review is focused solely on aroma compounds.

## 2. Precursors of Volatile Sulfur Compounds

### 2.1. Amino Acids

The proteins that build every living organism consist of 20 standard or canonical amino acids—aliphatic, which are the most prevalent, aromatic, and heterocyclic. Nine of them must be supplied through the diet, as they cannot be produced in the human organism [29]. Amino acids control the nutrostat system, take part in regulating blood pressure, can be used as an alternative source of fuels and biosynthetic materials, and show crucial importance as antioxidants and protective agents, especially when they contain sulfur in their structure [30,31,32].

**Figure 2:**
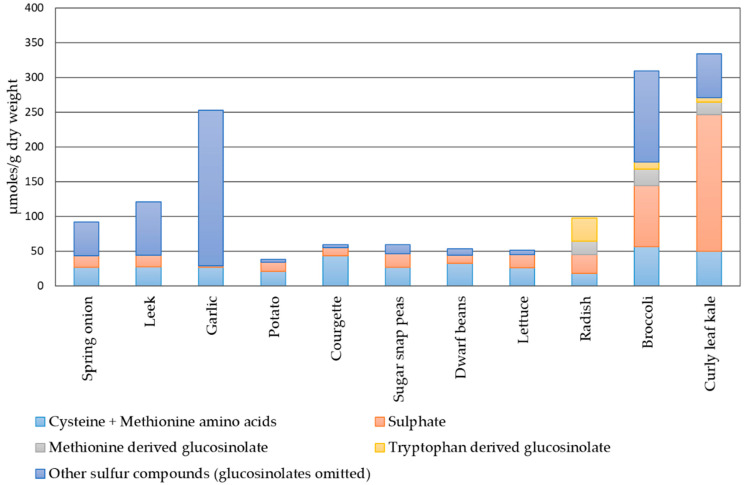
Sulfur distribution in selected vegetables based on [35].

Amino acids that have a sulfhydryl group in their structure—sulfur amino acids (SAAs)—are represented by cysteine and methionine [33]. The main function of cysteine in plants is maintaining an inorganic sulfur level obtained from the environment. This amino acid is the sole sulfide donor needed for the biosynthesis of crucial biomolecules, in particular, antioxidants (glutathione), cofactors, vitamins, or defense compounds—glucosinolates, thionins, phytoalexins. Methionine belongs to the group of essential amino acids, and it determines the development and is potentially responsible for the biosynthesis of growth-regulating substances (auxins, brassinosteroids, and cytokinins) [34]. It is also an important precursor to the biosynthesis of carnitine, cysteine, lecithin, taurine, and phospholipids. SAAs, such as methionine and cysteine, are the principal source of sulfur in the human diet supplied with food of plant origin. It was estimated that approximately 70% of sulfur obtained from plant products is accumulated in amino acids [1]. However, alliaceous and cruciferous vegetables have lower sulfur levels accumulated in amino acids in favor of specialized metabolites (Figure 2).

### 2.2. Glucosinolates

**Figure 3:**
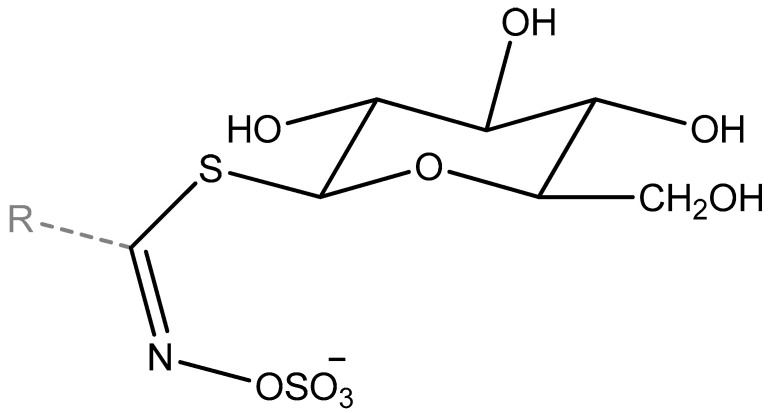
Structure of GLSs; R expresses side chain.

Glucosinolates are amino acid-derived secondary pant metabolites containing a sulphate and a thioglucose moiety (Figure 3). Their origin from particular amino acid structures determines their classification into aliphatic (usually methionine derived), aromatic (phenylalanine and tyrosine derived), or indolyl (originating from tryptophan). The GLSs biosynthesis comprises three stages: (i)—synthesis of chain elongated aminoacids; (ii)—glucone common to all glucosinolates addition and; (iii)—side chain modification [36]. In the process of side chain elongation, methionine can be elongated to homo, then dihomo, and finally trihomo-methionine. The formation of glucone moiety starts from aminoacids conversion into oximes. Starting points for oximes formation are tyrosine, phenylalanine, methionine (with potential chain extension), and tryptophan. This yields the basic type sidechains, as listed in Table 2. The oximes are oxidized via aci–nitro compound, which serves as an acceptor for a thiol donor (preferably cysteine). The activated form of glucose (UDPG, uridine diphosphate glucose) is incorporated forming desulphoglucosinolate in S-glucosylation, transferred into glucosinolate in the presence of 3’-phosphoadenosine-5′-phosphosulfate [36,37]. For a detailed description on GLS biosynthesis, one can refer to the thorough review in [38].

GLSs are naturally occurring compounds found in 16 dicotyledonous families. As mentioned above, significant amounts of GLSs occur in plants of the *Brassicaceae* family, though they are also present at a high level in the *Capparaceae* and *Caricaceae* families. By 2012, around 132 natural GLSs were documented [39]. The content of GLSs in the plant depends on plant organs, species, and vegetable development stage, for example, broccoli sprouts have 10–100 times higher concentrations of glucoraphanin, in contrast to mature broccoli. 

GLSs are non-volatile compounds and their taste is associated with cruciferous vegetables. Though GLSs are non-odoriferous, their taste influences the characteristic flavor of Brassica vegetables. It is generally approved that bitterness is related to the presence of these sulfur-containing compounds in the plant [40]. Nevertheless, there is currently limited research focusing on the sensory evaluation of pure compounds and estimation of their impact on overall flavor assessment. The sensory evaluation of GLSs is problematic due to the personal abilities of panelists (non-tasters, bitter blind), limited quantities, or the lack of suitable and pure standards that could be used in taste sensory tests. These reasons contribute to a lack of taste knowledge for most of the studied GLSs [41]. The existing studies showed that there are differences in the perception of GLSs bitterness. Taste evaluation of four GLSs indicated that most taste panelists (71% for sinigrin and 79% for gluconapin) found a bitter taste. However, in the case of glucobrassicin and progoitrin, only 21% and 9% of panelists, respectively, stated a bitter taste. A bitter mouthfeel of GLSs may be reduced by the application of debittering processes, such as selective breeding, non-bitter cultivars, or customized growth conditions [42].

**Table 2:** Chemical structure of glucosinolate side chain [43,44].

Name	Structure of Side Chain	Systematic Name	Type of Glucosinolate
Glucobrassicin	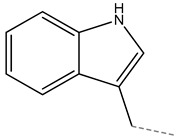	3-indolylmethyl glucosinolate	Indole
Glucotropeolin	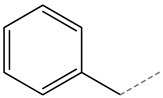	benzyl glucosinolate	Aromatic
Glucoraphanin	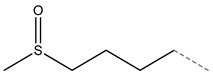	4-methylsulfinylbutyl glucosinolate	Aliphatic
Progoitrin	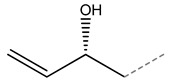	2(R)-2-hydroxy-3-butenyl glucosinolate	Aliphatic
Sinigrin	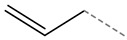	allyl glucosinolate	Aliphatic

According to the scientific literature, the great importance on bitter taste sensation has the concentration of plant constituents with a sweet mouthfeel, e.g., sugars and amino acids. In reference to the first, a sensation of bitter taste in cruciferous vegetables can be reduced by sweetness caused by the presence of free sugars, such as fructose, galactose, glucose, or sucrose. Vegetables differ in the ratio of free sugars to GLSs; higher ratio value results in desirable consumer preferences. Amino acids, such as alanine, proline, serine, and threonine, induce a sweet taste against leucine and valine, which are responsible for bitterness mouthfeel [42]. Some research showed an association between the content of sweet-tasting amino acids and reducing the effect in a bitter taste. However, the correlation between amino acid levels and GLSs content in vegetables requires more attention and additional research [41].

The moiety of GLSs consist of an unchanging part β-D-thioglucoside-N-hydroxysulfates and side chain, which is different for each GLS because it is a derivative of one of eight amino acids (Figure 3). GLSs are divided, according to the amino acids from which the side chain is formed, into aliphatic (Ala, Leu, Ile, Met, Val), aromatic (Phe, Tyr), and indole (Trp) GLSs; nevertheless, side chain is generally subjected to many modifications, such as acylation, desaturation, elongation, glycosylation, hydroxylation, and O-methylation [43]. The side chain variety of selected GLSs is shown in Table 2. The side chain of GLSs determines the character and structure of volatile compounds released after GLSs enzymatic or thermal degradation.

The enzyme myrosinase catalyzes the breakdown of GLSs. To initiate the hydrolysis reaction, it is necessary to disrupt the plant tissue (chewing, chopping, crushing, cutting) because GLSs are found in the cytoplasm, whereas the enzyme is stored in vacuoles. The first stage of hydrolysis degradation is the cleavage of the β-D-glucose molecule and obtaining an unstable intermediate product called an aglycone. Aglycone is subject to a spontaneous Lossen rearrangement, causing detachment of sulfate ion and depending on many factors (e.g., pH, presence of Fe^2+^ ions, or specialized proteins) described below, obtaining various aroma sulfur volatile compounds, such as epithionitrile, ITC, nitrile, oxazolidine-2-thione, and thiocyanate [6,38,45].

## 3. Biosynthesis of Sulfur Volatiles in Vegetables

The formation of VSCs in vegetables has been intensively explored in Allium vegetables. The second group of vegetables with important and abundant VSCs described is *Brassicaceae.* These two groups of vegetables dominate, though S-methylmethionine derived compounds are formed in asparagus and other vegetables. The Allium genus comprises about 700 species, of which garlic (*Allium sativum*) and onion (*Allium cepa*) are the most important vegetables appreciated mainly for their unique flavor. 

The formation of Allium flavor include: (i) enzyme-mediated degradation of non-volatile precursors; (ii) secondary reactions of primary flavor compounds, and; (iii) thermal degradation of precursors. Cysteine as a sulfur containing amino acid plays a crucial role in formation of flavor compounds of Allium vegetables along with glutamine, as (+)-S-alk(en)yl cysteine sulfoxides (CSOs) and their γ-glutamyl peptide (γGPs) are the main non-volatile, odorless precursors [46]. Detailed review papers on biosynthesis of flavor compounds and their precursors have been published [46,47,48]. 

Garlic, onion, and other members of the Allium contain 1–5% dry weight of nonprotein SAA secondary metabolites. The disruption of the cell results in the release of allinase (C-S lyase) are present in vacuole and the reaction with S-alk(en)yl-L-cysteine S-oxides (volatile compounds precursors) located in the cytoplasm [49]. Four sulfoxides occur in Allium species: S-2-propenyl-L-cysteine sulfoxide (alliin, ACSO), S-(E)-1-propenyl-L-cysteine sulfoxide (isoalliin, 1-PeCSO), S-methyl-L-cysteine sulfoxide (methiin, MCSO, also present in Brassica vegetables), and S-propyl-L-cysteine sulfoxide (propiin, PCSO). Differences in flavor within Allium is caused in various amounts of these precursors [47]. As summarized by Block, based on experiments with labelled ^35^SO_4_^2-^ fed to onion plants, the sulfate is reduced and assimilated into cysteine in the chloroplasts. Then incorporation of glutamic acid yields γ-glutamylcysteine, which reacts with methacrylic acid (from valine) to yield γ-glutamyl-S-2-carboxypropylcysteine, which can undergo sequential decarboxylation to γ-glutamyl-S-1-propenylcysteine, Subsequently oxidation to γ-glutamyl-S-1-propenylcysteine-S-oxide takes place and it is cleaved by γ-glutamyl transpeptidase [EC 2.3.2.1] to S-(E)-1-propenylcysteine S-oxide [47]. 

**Figure 4:**
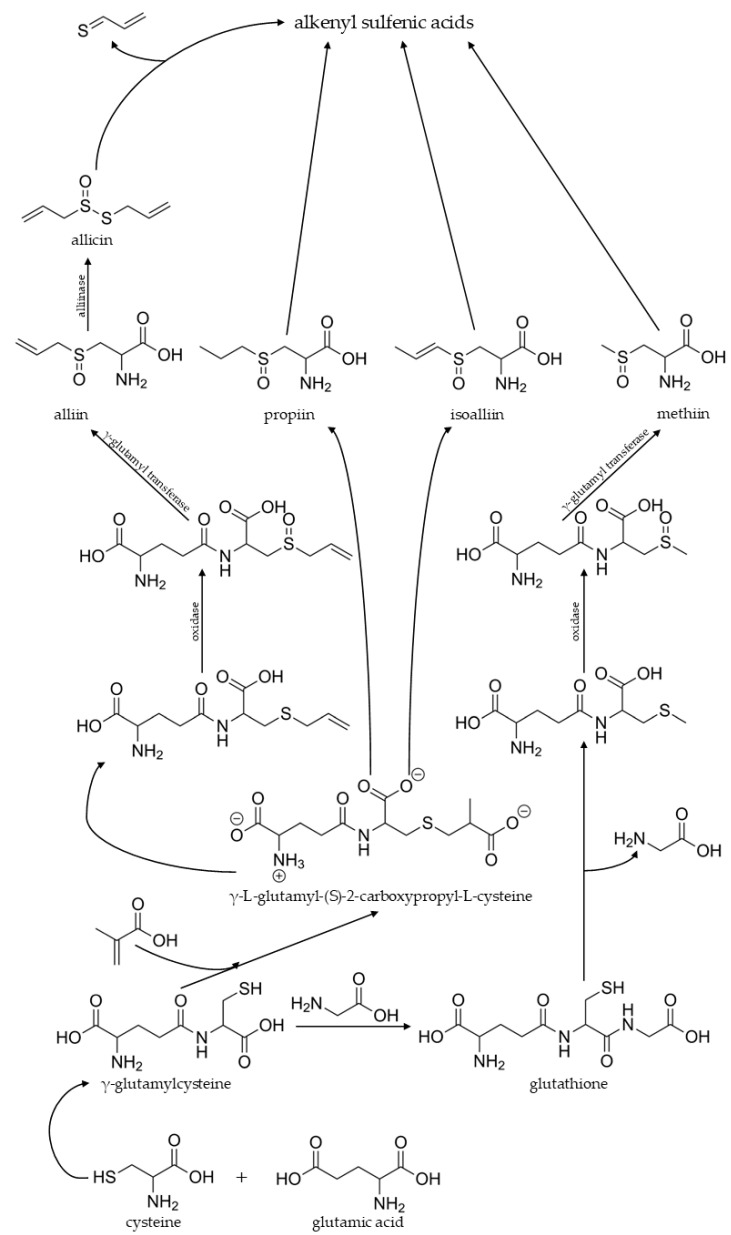
Initial stages of Allium flavor compounds formation by enzymatic reactions. Based on [47,48,49,51].

The main enzymes, involved in the formation of Allium volatiles—allinases [EC 4.4.1.4]—are α,β-eliminating lyases that catalyze the decomposition of the above-mentioned S-oxides to ammonium pyruvate and allicin and its homologues. Sulfenic acids, which are highly unstable, are key enzymatically formed intermediates in Allium chemistry. The initial stages of sulfenic acids enzymatical formation has been summarized on Figure 4.

It is assumed that the flavor components of garlic and onion mainly include S-allyl cysteine sulfoxide (allicin); S-allyl-cysteine; and the sulfides of diallyl-, methyl allyl-, and dipropyl mono-, di-, tri-, and tetrasulfides, all being produced by sulfur-containing precursors—mainly S-alkenyl cysteine sulfoxides (ACSO) through an enzyme-mediated degradation process [48]. Allicin (C_6_H_10_OS_2_), a key molecule in garlic flavor, is responsible for the typical smell and taste of freshly cut or crushed garlic. It accounts for 70% (w/w) of the total thiosulfinate compounds present/formed during the crushing of garlic cloves [50]. Allicin is very unstable and can easily be converted to diallyl sulfide (DAS), diallyl disulfide (DADS), diallyl trisulfide (DATS), and diallyl tetrasulfide (DATTS).

Due to the instability of primary flavor compounds of Allium, they can decompose or undergo condensation to secondary ones—these derived from alk(en)yl thiosulfinates. Allicin is the best example, as it undergoes degradation to a variety of sulfides (mainly DADS). It is sensitive to pH (>11), solvents, and temperature. At temperatures exceeding 50 °C, it decomposes rapidly [52]. It was investigated that heating S-methyl-L-cysteine and S-methyl-L-cysteine sulfoxide in a model system with varying water content and temperatures resulted in formation of volatile breakdown products of these precursors [53] that can contribute to the thermally processed Brassica and Allium vegetables flavor. The predominant volatile was dimethyl sulfide. Dimethyl trisulfide, dimethyl thiosulfinate, dimethyl thiosulfonate were also detected as products of S-methylcysteine sulfoxide (MCSO) degradation. Dimethyl trisulfide was proposed as an off-odorant of overcooked vegetables [53]. The data on the thermal degradation of flavor precursors in garlic and onion was collected by Li and coworkers, and, according to this, the main degradation products of MCSO are dimethyl disulfide and, to a lesser extent, dimethyl trisulfide (after heating at 120–160 °C); for PCSO—dipropyl disulfide and dipropyl trisulfide and propylthiol; and for alliin—diallyl sulfides (mono-, di-, tri-, and tetra-), methyl sulfides, 2-acetylthiazole, and other cyclic compounds (2,5-dimethyl-1,4-dithianes, 2-methyl-1,4-dithiepae, and others) [48].

Sulfur containing volatile compounds formed in Brassica originate mainly from the hydrolysis and degradation of their precursors—GLSs, as discussed in the previous chapter. The detailed pathways for the formation of VSCs being the products of GLSs degradation were discussed in [5].

**Figure 5:**
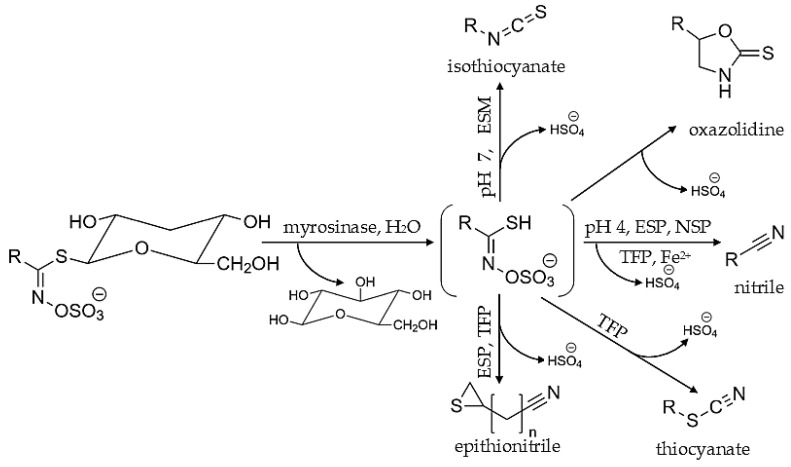
Formation of main groups of volatile compounds as a result of GLSs hydrolysis in Brassica vegetables; ESP−epithiospecifier protein; ESM−epithiospecifier modifier protein; NSP−nitrile-specifier proteins; TFP−thiocyanate-forming protein; R−side chain [6,54].

The breakdown of GLSs aglycone is strictly contributed to reaction conditions. The receiving of ITCs is favored when the reaction environment is neutral or slightly acidic (pH range from 5 to 7), which corresponds to plant tissue. ITC molecules consist of an isothiocyanate group (-N=C=S) stuck to carbon chain (-R) that can be represented by a general formula R-N=C=S (Figure 5).

The formation of methanethiol and dimethyl trisulfide in disrupted cabbage tissue from the reaction of thiosulfinates and thiosulfonates and hydrogen sulfide following the action of CSO lyase was proposed, whereas dimethyl sulfide was proposed to originate from chemical disproportionation of methyl methanethiosulfinate [51]. Dimethyl disulfide, often detected as major VSC in the headspace of freshly disrupted cabbage tissue, is considered a secondary product of C-S lyases hydrolysis of S-methyl-L-cysteine sulfoxide (MCSO), the primary reaction product being methanesulfonic acid, which dehydrates to more stable methyl methanethiosulfinate, which is disproportionate to dimethyldisulfide. Alternative mechanism could be proposed involving methanethiol oxidation to dimethyl disulfide in the presence of air. The formation of dimethyl trisulfide was postulated via reaction of dimethyl disulfide obtained after C-S lyase action from S-methyl-L-cysteine sulfoxide with elemental sulfur, or reaction of methanesulfonic acid and hydrogen sulfide [51]. 

**Figure 6:**
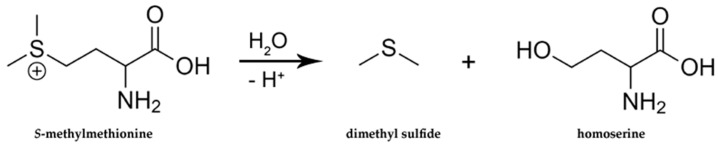
Thermal degradation of S-methylmethionine.

S-methylmethionine was identified in asparagus by Challenger and Hayward (1954), and it has been shown that it undergoes decomposition during food storage/thermal treatment into dimethyl sulfide being a part of boiled asparagus, processed tomatoes, corn, and beer flavor (Figure 6). The S-methylmethionine has been found in a range of 2.8–176 mg/kg in tomato, cabbage, turnip cabbage, beetroot, celery, and leek [55].

Sulfur containing acids were identified in asparagus and characterized in the 1970s, as precursors of aroma compounds [56,57]. They were 1,2-dithiolane-4-carboxylic acid (asparagusic acid), 3,3-dimercaptoisobutyric acid (dihydroasparagusic acid), and S-acetyldihydro asparagusic acid. It was found that sulfur compounds are formed in asparagus in the intact plant cells, which is an exceptional case in the formation of sulfur compounds, which are usually formed by enzymatic reactions. In asparagus, 13 sulfur containing acids were detected (of which asparagusic acid is predominant) and three esters (methyl 1,2-dithiolane-4-carboxylate being the dominant one). After performing [U-^14^C], labelled experiments valine was postulated as a starting molecule that is transformed via corresponding oxo acid, isobutyric acid, and methacrylic acid into 3-mercaptoisobutyric acid, 3-methylthioisobutyric acid, and, to a lesser extent, asparagusic acid [56]. In the cooking process, asparagus yields compounds belonging to different chemical classes: sulfur compounds (17), pyrroles, pyridines, pyrazines, furanes (25), aldehydes (12), ketones (15), alcohols (26), phenols (11), lactones (5), acids (10), and dimethyl sulfide and methyl 1,2-dithiolane-4-carboxylate (3000 ppb and 7000 ppb, respectively) [57].

Sulfides are formed in vegetables from the methionine or cysteine pathway or transforming other sulfur substrates, e.g., ITC rearrangement reactions [58]. For example, vegetables belonging to the *Amaryllidaceae* family include *S*-alk(en)yl-L-cysteine sulfoxides in n the cytosol of storage mesophyll cells, whereas alliinase is sequestered in the vacuole of vascular bundle sheath cells vacuoles of plant cells [59]. The mechanism of sulfide formation is similar to that of the GLS-myrosinase system. Tissue disruption is required to allow the enzyme to contact the substrate and a series of unstable products is then created. Sulfenic acids are converted into thiosulfinates and, finally, into a complex mixture of compounds, in which mono-, di-, and polysulfides predominate [60]. Some studies show a close relationship between sulfide formation and the raised temperature necessary for industrial processes, for example, in preparing tomato paste or cooking cabbage [27]. The extension of the influence of industrial processes will be discussed in the following paragraphs.

**Figure 7:**
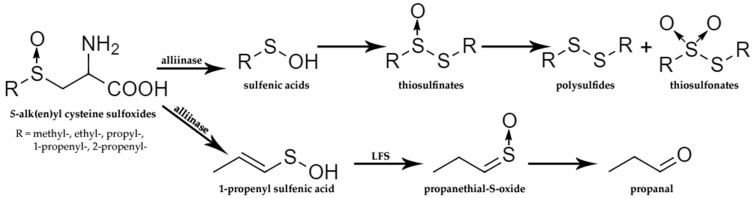
Transformations of cysteine sulfoxides (CSOs) after tissue disruption. LFS—lachrymatory factor synthase based on [61].

After tissue disruption alliinase reacts with ASCOs and cleaves their C–S bond, obtaining sulfenic acid, pyruvic acid, and ammonia. Notoriously unstable sulfenic acids undergo additional chemical reactions according to the non-enzymatic pathway (self-condensation resulting in thiosulfinates) and enzymatic mechanisms (for example 1-propenyl sulfenic acid lachrymatory factor synthase (LFS) leads to propanethial S-oxide and, finally, in the presence of water forms propanal). Concisely, a series of sulfur organic compounds contributing to aroma creation (odor sensation, mouthfeel) and bioactive properties of *Allium* plants are spontaneously obtained from sulfenic acids in the absence of the enzyme that catalyzes the conversion of sulfenic acids [59,61]. Figure 7 shows the formation of sulfur compounds from CSOs, indicating the pathway to lachrymatory factor (in onion, propanethial-S-oxide), as well as a simplified route, leading to polysulfides and thiosulfonates.

## 4. Main Groups of Volatile Sulfur Compounds

### 4.1. Isothiocyanates

In recent years, knowledge of ITCs has increased significantly. As a result of the growing interest in human health and research, it was possible to learn about many of the desired properties of these compounds. Most studies of ITCs properties are a concern to their chemopreventive and chemotherapeutic action [62]. Moreover, antimicrobial properties have been demonstrated, which can be used in agriculture for plant protection and food preservation as well [63]. Anti-inflammatory effects have been examined, respectively, for example, allyl ITC [64] and benzyl ITC [65].

**Figure 8:**
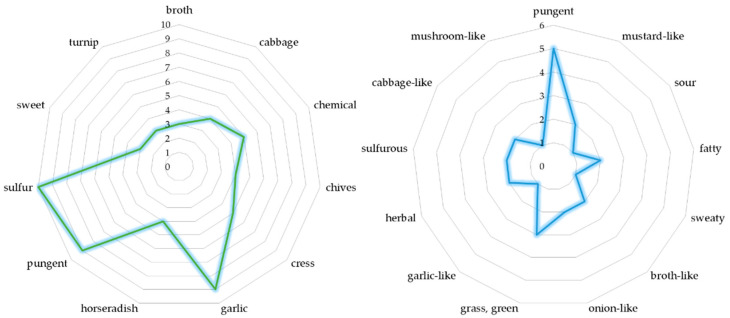
The most common odor descriptors for isothiocyanates (green) [68], nitriles, and epithionitriles (blue) [26,61,62,63,64] found in vegetables.

Although GLSs are non-volatile and odorless precursors of their hydrolysis products ITCs [66], the pungent flavor of Brassica vegetables is associated with the presence of ITCs [67]. ITCs have specific aroma of a wide variety. There is a relationship between chemical structure and the odor threshold value. According to the increasing number of carbon atoms in aliphatic molecules, odor threshold value decreases, with the exception of ethyl ITC, which has a higher odor threshold value than methyl ITC. The chemical structure of ITCs (e.g., aliphatic saturated and unsaturated, aromatic, branched, cyclic, and miscellaneous) determines not only odor thresholds but odor quality too. Quite the non-obvious quality of the smell that ITCs described were sweet, fruity, floral, herbs, mushroom, or even fish descriptors. Of all recorded odor descriptors, the most frequent for ITCs are sulfur, garlic, and pungent smell [68] (Figure 8). Nevertheless, the sensory evaluation of ITCs odor is still limited, and there are not many studies that would allow us to compare odor quality and thresholds.

There are generally very limited, if any, data on the taste of ITC. They occur naturally in a complex food matrix, often in a large number. Insufficient information on bi-modal sensory activity of ITC, combining both their aroma and taste leaves many questions on their role in flavor formation unanswered. The quantitative determination of GLSs and ITCs separately and statistical analysis showed correlations only between some compounds and bitter taste [41].

### 4.2. Nitriles and Epitionitriles

Nitriles and epitionitriles are produced by the rearrangement of unstable aglycone in the presence of specified cofactors, such as nitrile specifier proteins (NSP), epithiospecifier proteins (ESP), or specific environmental conditions [69,70]. The presence of nitriles and epithionitriles are significantly connected with edible parts and the stage of growth for crops from the *Brassicaceae* family. ITCs are the most abundant GLSs; hydrolysis produces edible parts, such as leaves in white cabbage, Savoy cabbage, or heads of Brussels sprouts, whereas nitriles and epitionitriles are mainly found in considerable concentrations in sprouts and seeds [71].

Although in scientific reports, occupational exposure to nitriles is commonly associated with numerous health disorders (e.g.,: cardiovascular, gastrointestinal, hepatic, neurologic, renal) [72], food borne nitriles also show cytotoxic and genotoxic potential [73]. The presence of nitriles in Brassica can be associated with health-promoting effects [74]. Compared to ITC and their bioactivity, relatively little is known about nitriles. Due to reduced beneficial health-promoting activities of nitriles, in comparison with ITCs, some strategies are used to select particular varieties of vegetables. They are characterized by either reducing the activity of ESP or increasing the activity of the native enzyme, myrosinase [70].

Nitriles as volatile compounds, in comparison to corresponding aldehydes, have comparable odors to corresponding aldehydes [75]. Nitriles and epithionitriles found in vegetables show diverse aromas from pleasant grassy, herbal, broth-like notes to unacceptable ones, such as pungent, sulfurous, or sweaty. Figure 8 summarizes the most common descriptors of nitriles and epithionitriles found in vegetables.

Taking into consideration that the taste of nitrile compounds is unknown, it is not possible to identify how nitrile derivatives influence taste in major vegetables, if at all. In the present day, we do not have data that indicate the bitter taste of nitriles and epitionitriles [41]. Therefore, further research is needed, in particular sensory analysis and taste evaluation of these GLS hydrolysis products.

### 4.3. Sulfides and Polysulfides

The presence of sulfide volatiles in food samples is commonly associated with an unpleasant and unacceptable odor. Indeed the cause of rejection of cauliflower is the presence of dimethyl trisulfide and dimethyl sulfide, among other odorants [76]. High concentrations of sulfides are present in vegetables from the *Brassicaceae* and *Amaryllidaceae* families [77,78]. Vegetables containing abundant organic sulfides in different species have strong antioxidant activities and these important bioactive compounds exhibit antibacterial and immune activities [79]. 

Sulfides play a crucial role in the creation of organoleptic properties of food, which is why many synthetic sulfides are commonly used as food flavors (more than 80 mono-, di-, and polysulfides) and additives in processed foodstuff [60]. Sulfides are also associated with the off-flavors of many other products, such as drinking water, alcoholic beverages, and juices [80,81,82,83]. The sensory properties of sulfides are similar to each other because these compounds are most often characterized by a pungent smell and a spicy taste [26,84,85].

### 4.4. Thiols and Miscellaneous Sulfur Compounds

Volatile thiols are mainly associated with off-flavors in various types of food and beverages [86,87]. Among the breakdown products of GLSs, thiols are an important group that determines the smell of vegetables, especially *Brassica* vegetables. Research shows that in raw broccoli, methanethiol and 1-pentanethiol are two aromatic active compounds with the highest flavor dilution factor. What is more, thiols can be oxidized to the corresponding sulfides [88]. Thiols were also found in green kohlrabi but were not the key flavor active compounds [89]. The regulation of thiol biosynthesis in plants means their redox status is related to plant stress tolerance; nevertheless, the biochemical pathways have not been completely explained so far [90].

Interesting aroma-active compounds that contain a sulfur atom in their structure are odorants found in truffles and shiitake mushrooms, in which volatile compounds are formed in the enzymatic and non-enzymatic way. The compounds responsible for the aroma of shiitake mushrooms come from the fatty acid pathway and the amino acid pathway. Linolenic acid and linoleic acid are the precursors of the fatty acid pathway. They are converted by enzymes—lipoxygenase, hydroperoxide lyase, and alcohol dehydrogenase—to generate aldehydes and alcohols (mostly C6, C8, and C9 volatiles). Multi-step oxidation and polymerization of sulfur-containing amino acids provide precursors, namely lentinic acid, which is a precursor substance to form heterocyclic sulfur volatiles in the presence of enzymes, in particular, γ-glutamyl transpeptidase and CSO lyase [91]. Sulfur volatiles obtained from shiitake mushrooms responsible for distinctive, sulfurous aroma is related to the presence of 1,2,4-trithiolane, 1,2,4,6-tetrathiepane, and lenthionine [92]. However, in truffles, the origin of sulfur-containing volatiles is complex and unclear at the same time. Aroma-active compounds might be derived from the truffle itself but also the microbial population residing in truffle-fruiting bodies. Organic volatile compounds are biosynthesized, mainly by sulfate reduction, amino acid, and fatty acid catabolism pathways [93,94].

An interesting flavor compound that has been found in many vegetables, as well as shiitake mushrooms and truffles, is methional. Biosynthesis begins with the amino acid methionine, which is converted to methional through Strecker degradation, in which intermediates in the Maillard reaction interact with the methionine. Methional as a volatile compound containing a sulfur atom in its structure is characterized by a low detection threshold, and the quality of its smell is defined as pleasant, reminiscent of a boiled potato. What is more, methional can be further converted into sulfide and disulfide compounds [95,96]. Table S1 (Supplementary Files) lists the main aroma sulfur compounds with their odor qualities, representing different classes found in vegetables and mushrooms. 

## 5. Sulfur Compounds as Key Odorants in Vegetables

**Figure 9:**
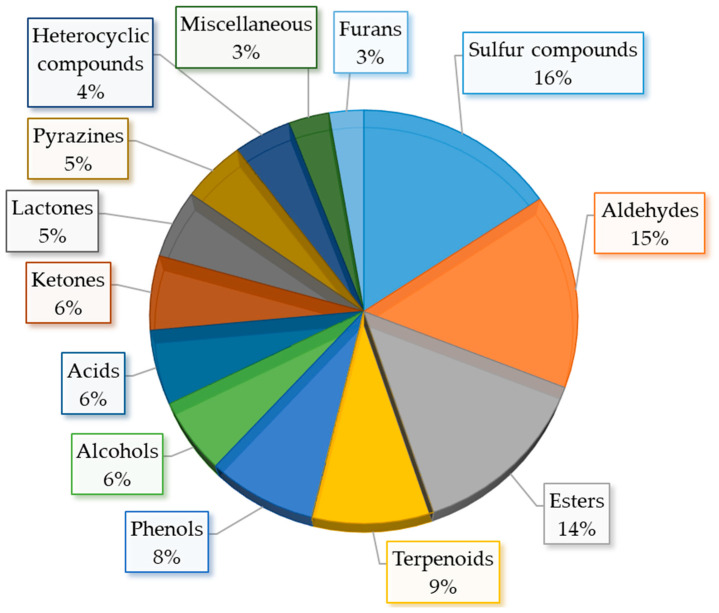
Key food odorants detected in food samples based on [97].

In the fundamental review on food key odorants, presence in various food products sulfur compounds are 16% of all (226) of them [97]. Along with aldehydes and esters, sulfur compounds form 45% of all key odorants (Figure 9). However, one has to remember that key odorants form roughly only <3% of all volatiles in foods. 

VSCs in food form a significant group of compounds, regarding their number and, what is probably more important, their odor thresholds and odor notes. As estimated for a piece of research conducted prior to 1991, a total of 633 VSC were detected in food [98], and one can presume that the number increased within last 30 years. 

Although in many publications VSCs are regarded as compounds of potential influence on the aroma of vegetables, there are not many papers devoted to elucidation of their key odorants. The use of gas chromatography—olfactometry (GC-O)—to detect aroma compounds of foods and methods to quantify the sensory sensation, started the sensomics approach to characterize foods [99]. After vacuum distillation - the most frequent technique being SAFE (solvent-assisted flavor evaporation), it involves the application of GC-O to detect odor active regions in chromatogram and then identify compounds with the highest sensory role in the creation of the overall aroma of a product. Usually to present the aroma importance of particular compounds, FD (flavor dilution) is determined by subsequent GC-O analyses of a serially diluted extract of aroma compounds (AEDA, aroma extract dilution analysis). Moreover, the concept of OAV value (defined as ratio of compound concentration to its odor threshold) is used in the characterization of odorants [99]. 

Literature data on key odorants in vegetables can be divided into early works on crucial sensory active compounds, especially in Allium vegetables; some works are on other vegetables, mainly Brassica, and there are works, especially in recent years, on compounds migrating in the cold pressing of rapeseed oils, especially after roasting seeds [69,100,101,102]. 

The early works on VSC presence and also their sensory properties were summarized by Boelens (1993), who provided data for onion, garlic, and leek, but also truffle, tomato, and potato. In raw onion, thiopropanal-S- oxide, as well as propyl methanethiosulfonate and propyl propanethiosuflonate with OT 1700 and 1500 ng/L for the latter two compounds, respectively, were detected. Dipropyl disulfide and *cis*- and *trans*-1-propenyl propyl sulfide contributed to the aroma of cooked onion. After heating, they tend to form dimethylthiophenes with a distinct fried onion aroma. The most important odorants in garlic were di(2-propenyl)disulfide and di(2-propenyl)trisulfide. In leek, compounds possessing leek aroma were propanethiol, methylpropylsulfide, methylpropyldisulfide, and also 3,4-dimethyl-2,5-dioxo-2,5-dihydrothiophene, which can possibly hydrolyze in water to yield hydrogen sulfide [98]. 

Several Brassica vegetables were investigated using chromatography and olfactometry. In cauliflower, the main compounds responsible for sulfur odor notes detected by GC-O were methanethiol, perceived by sniffers as “sulfur, cooked cabbage”, dimethyl sulfide (DMS) described as “cauliflower”, allyl isothiocyanate described as “black mustard-like and pungent”, and dimethyl trisulfide (DMTS) with the odor note as “sulfur, cauliflower, cabbage”. Of 63 odor active compounds, 13 were sulfur compounds [103]. Key odorants of raw and cooked kohlrabi (*Brassica oleracea var. gongylodes* L.) were analyzed, and a total of 55 odor active compounds were detected and identified using GC-O. Twenty-eight compounds with the highest FD were quantified and OAV were determined. Of eight compounds with the highest OAV, five were sulfur compounds (dimethyl trisulfide, methyl 2-methyl-3-furyl disulfide, and three ITCs—1-isothiocyanato-3-(methylsulfanyl)propane, benzyl isothiocyanate, and 1-isothiocyanato-4-(methylsulfanyl)butane). The same compounds formed the backbone of both raw and cooked kohlrabi; differences were noted in OAV values and minor compounds [89]. Raw and cooked broccoli was subjected to GC-O and AEDA to assess the main odorants. Among 30 odor active compounds in raw broccoli, 19 were sulfur-containing ones. They were also characterized with the highest FD values. Two compounds with the highest FD were methanethiol and 1-pentanethiol (FD 1024). They were followed with dimethyl sulfide, dimethyl trisulfide, 2-methyl methanethio sulphonate, 4-methylpentyl isothiocyanate, hexyl isothiocyanate, dimethyl tetrasulfide, and 3-methylthiopropyl isothiocyanate (all FD 256). In contrast, cooked broccoli florets had only nine odor active compounds, among which dimethyl sulfide and dimethyl trisulfide were detected [88]. For *Brassica rapa* cv. Yukina, 12 odor active compounds were detected among 50 volatiles using GC-O. Among them, eight compounds contained sulfur. The highest FD factors were obtained for dimethyl tetrasulfide, 3-phenylpropanenitrile (FD = 64), methional (FD = 32), and dimethyl trisulfide (FD = 16) [101]. 

For fermented Brassica vegetables, GC-O was used to assess the odor active compounds present in Kimchi. It is a traditional Korean fermented vegetable product in which Chinese cabbage is a main ingredient. Minor components can be red pepper, garlic, ginger, and fish sauce. For a Kimchi, in which Chinese cabbage was 86.1%, which also included garlic (1.4%) and <1% of leek, green onion, ginger, and carrot, 23 sulfur compounds were detected among 160 volatiles. The highest abundances were noted for DADS, methylallyl disulfide, dimethyl trisulfide, and dimethyl disulfide, whereas the highest odor intensities were noted for dimethyl trisulfide, DADS, and DATS [104]. In another fermented vegetable called Yongchuan Douchi (fermented soybean)—a traditional Chinese product—49 aroma active compounds were detected by GC-O. Twenty-two compounds were characterized by determining FD and OAV. Among these 20 key odorants, two sulfur compounds were playing an important role, where dimethyl trisulfide was characterized by an OAV of 8818—the highest OAV. For 3-(methylthio)propionaldehyde OAV was 229 [105]. Tempeh prepared from soy, fermented with *R. oligosporus* for 1 and 5 days, showed 21 odor active compounds. Among the three were sulfur compounds—dimethyl sulfide, dimethyl trisulfide, and 3-(methylthio)propanal. After 5 day fermentation, 3-(methylthio)propanal was characterized by the highest OAV (680) of all 21 compounds. Dimethyl trisulfide was also among the seven most potent odorants with OAV 120 [106]. In fermented fresh garlic, known as black garlic in Asia, 52 aroma compounds were detected after SAFE and SPME extraction and when assessed using FD, 24 compounds with the log2FD >2 were selected. Nine of them were sulfur compounds with the highest log2FD values noted for allyl methyl trisulfide (8), 2-vinyl-4H-1,3-dithiine (6), DATS (5), 3-vinyl-1,2-dithiacyclohex-4-ene (5), and 3-(methylthio)propionaldehyde. The highest OAV value calculated for black garlic were noted as 5-heptyldihydro-2(3H)-furanone (536) followed by DADS (188) and (E,Z)-2,6-nonadien-1-ol (134) [107]. 

Aged garlic extract (ethanolic, AGE) aged for more than 10 months was compared to fresh garlic. When AEDA was performed, fresh garlic compounds with the highest FD factor were 2-vinyl-4H-1,3-dithiin (FD 65,536), followed by S-methylmethanethiosulfinate (FD 256), 3-vinyl-4H-1,2-dithiin, methional (both FD 128), diallyldisulfide, allyl methyl trisulfide (FD 64), and allyl mercaptan (FD 32). All identified odorants of fresh garlic were sulfur compounds. Moreover, 2-vinyl-4H-1,3-dithiin was the main odorant in AGE, however, it was followed by phenolic compounds absent in fresh garlic [24].

Dimethyl trisulfide was detected by GC-O as an odor active compound in dried bell peppers [108]. Methional was characterized with an FD of 512 and was detected in Hungarian sweet bell pepper powder, however, the most intense odorants in it were β-ionone (FD 32768), furaneol (FD 16384), 2 and 3-methylbutanoic acid, and sotolon (FD 8192). The role of methional as an odor active compounds in Moroccan sweet bell pepper powder was more significant [109]. In red bell pepper, a secondary alkanethiol—2-heptanethiol with low OT (10 µg/L)—was detected [110]. Several sulfur compounds were detected in packed rocket leaves close to expiration date by GC-O: methanethiol, dimethyl sulfide, dimethyl disulfide, and 2,4-dithiapentane [111]. Interesting sulfur-containing acids were characterized in asparagus, and in cooked asparagus, dimethyl sulfide and methyl 1,2-dithiolane-4-carboxylate were predominant [56], but their role in the formation of asparagus flavor has not been elucidated by sensomics. The odorants of cooked asparagus explored by GC-O and Charm analysis revealed 3-(methylthio)propanal and S-methyl thioacetate as important odorants, though the highest Charm values were noted for 2-methoxy-3-isopropyl pyrazine [112]. Sulfur compounds also play an important role in the aroma of truffles. When aroma active compounds of white alba truffles (WAT) were compared to burgundy truffles (BT), among the 56 odorants obtained from SAFE extracts, 5 were sulfur compounds: bis(methylthio)methane, dimethyl trisulfide, 3-(methylthio)propanal, 3-(methylthio)propanol and 1,2,4-trithiolane. Their sensory importance was high: for WAT, the compound with the highest FD factor was 3-(methylthio)propanal (4096), followed by bis(methylthio)methane (FD 1024). Interestingly, the last compound played a minor role in the aroma of BT when considering FDs. In the headspace, dimethyl sulfide and methanethiol were also detected. When OAV was calculated for key aroma compounds the highest value was observed for bis(methylthio)methane (817,000) in WAT, though not quantifiable for BT. Moreover, 3-(methylthio)propanal was characterized with very high OAV values (807 and 500), respectively [113]. 

The roasting process of rape seeds results in a grand increase in sulfur compounds responsible for aroma. When expressed as OAV, values for 2-furanmethanethiol in roasted rapeseeds was 14,200, followed by dimethyl trisulfide (13300) increased from unquantifiable values in raw rapeseed. Moreover, very high OAV were noted for methanethiol (1160), dimethyl sulfide (962), and 3-(methylthio)propanal (54). Among the 10 compounds with the highest OAV values, 5 were sulfur compounds. Similar results in profile of sulfur key odorants were noted for roasted mustard seeds, in which 2-furanmethanethiol was also the most pronounced odorant with an OAV of 36,300 [102]. When native, cold-pressed rapeseed oil was investigated using the sensomics approach, 54 aroma active compounds were noted, among them 47 were identified. Dimethyl trisulfide (FD 128) and 2-propionylthiazole (FD 512) were two sulfur compounds with high importance of flavor creation, though the highest FD values were noted for 2-isopropyl-3-methoxy pyrazine (IPMP) and 2-isobutyl-3-methoxypyrazine (IBMP) (both FD of 20148). Using HS-AEDA, dimethyl sulfide was also detected (FD 4). When OAV was calculated for odorants characterized by FD, the highest value was for IPMP (330), followed by dimethyl trisulfide (37) and dimethyl sulfide (37) [114]. Dimethyl trisulfide was one of the compounds responsible for fusty/musty defects in cold-pressed rapeseed. The FD values for this compound in fusty/musty rape seeds (2048) and oil obtained from these seeds (512) exceeded this in the control oil (128). When expressed in OAV, dimethyl trisulfide in fusty/musty oil (1900) and seeds (2900) exceeded the OAV for control oil (37) even more [115]. Pollner and Schieberle analyzed key odorants in cold pressed rapeseed oils from unpeeled and peeled seeds and among 60 compounds with FD > 2 dimethyl sulfide and dimethyl trisulfide were detected (FD 2 and 32, respectively, in oil from unpeeled seeds and FD 128 and 16 in oil from peeled seeds). The OAV values for DMS in commercial rapeseed oils (from peeled and unpeeled seeds) were as high as 480 and in 6 out of 10 samples were >200 [116]. Dimethyl sulfide was the key odorant in rapeseed oil obtained from traditional and high oleic rapeseed varieties, with dimethyl trisulfide also detectable [117].

In rapeseed oil obtained from microwaved seeds, four thiols with low odor thresholds were identified: 2-methyl-3-furanthiol (OT 0.481 µg/L), 2-furfurylmethanethiol (OT = 0.061 µg/L), phenylmethanethiol (OT 0.029 µg/L), and 3-sulfanyl-1-hexanol (OT 0.006 µg/L). The OAV in various rapeseed oils ranged from 2–28, 56–626, 104–3589, and 213–7565, respectively [118].

## 6. Influence of Vegetable Processing on Sulfur Compounds

Industrial processes are an important element in food production because they directly influence the preservation of its quality, extend the shelf life, or reduce in transport mass [119]. Dehydration and fermentation as preservation methods were utilized for centuries not only to preserve and extend the shelf life of food products but also to impart new, attractive sensory properties. Thermal processes are utilized due to the reduction in microbial population, destroying native enzymes and finally rendering food more palatable. Conversely, the main function of freezing technologies is the preservation of food as a result of lowered water activity and reduced rates of chemical reactions [120]. Nevertheless, there is no doubt that industrial processes have a considerable impact on the aroma of processed products, in particular, vegetables and mushrooms. The prudent choice of appropriate food processing methods from farm to consumer can guarantee that the health-promoting properties of specific bioactive compounds are preserved [121]. The research on quantitative aspects of flavor changes, involving especially sulfur compounds is limited. The majority of papers published focus on GLSs. A few selected industrial processes are presented in the following subsections.

**Figure 10:**
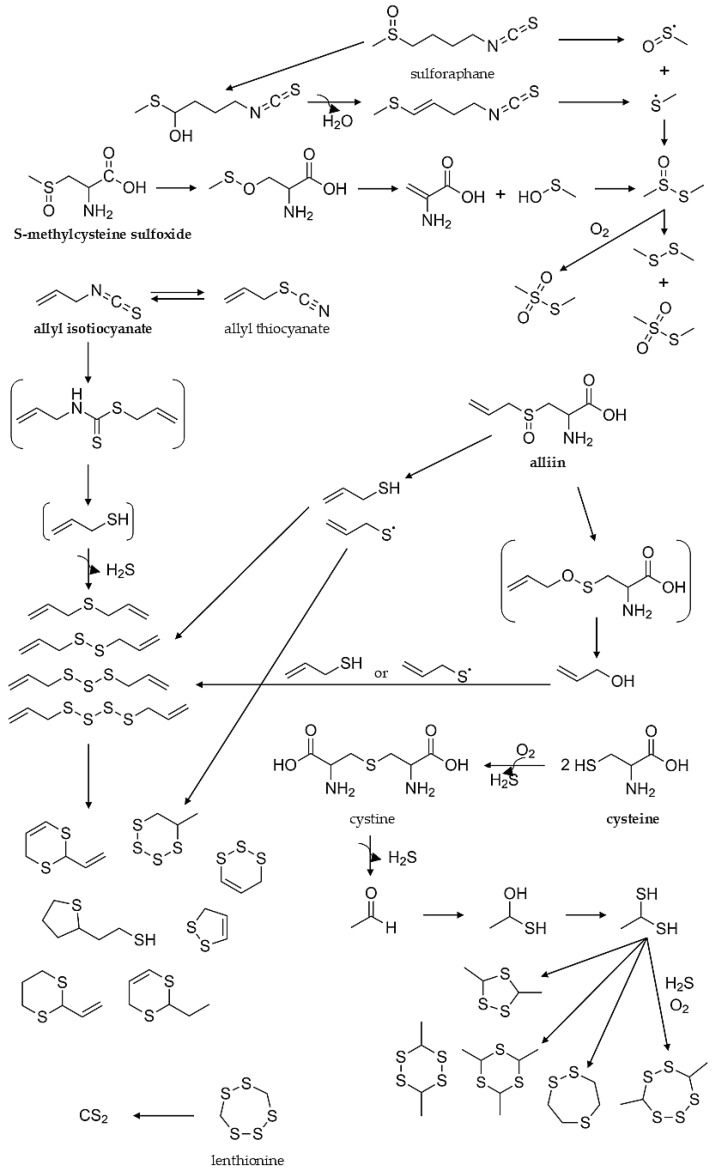
Thermal degradation of sulfur compounds in vegetables and mushrooms [122,123,124,125,126,127,128].

Many VSCs are formed during a variety of industrial processes, in particular, thermal processes. Figure 10 shows the thermal degradation and the production of the most important groups of VSCs, such as GLSs, ITCs, alliin, thiols, sulfides, polysulfides, lenthionine, and sulfur-containing flavor compounds, derived from SAA as cysteine and methionine. This makes the influence of high temperatures a substantial factor in creation, as well as stability of VSCs. Thermal lability of many sulfur compounds contributes to dynamic changes in mutual proportions of volatile compounds in products and flavor changes in it.

### 6.1. Drying

Drying is probably the oldest method of food preservation. Today, there are many drying options: combined convective hot-air and non-thermal drying involve other technologies, such as reduced pressure, ultrasound, pulsed electric field, and ultraviolet technologies [119]. Vegetables and other food extracts can be used as additives to commercial food products [129].

Among various drying techniques (convective drying, freeze-drying, vacuum microwave drying, combined drying) of shiitake mushrooms, freeze-drying showed the best results in terms of volatile compounds content and sensory parameters [130]. The spray freeze-drying (SFD) method, first introduced as a new method for biopharmaceutical powder preparation, was used to produce the aromatic powder obtained from shiitake mushrooms. It turned out that, as a result of SFD, the content of volatile and aroma-active compounds in food was reduced. Concerning sulfur compounds, the percentage of recovery ranged from 30.9 to 68.3%. Other important key odorants showed similar or higher recoveries. The greatest losses after the SFD of volatile compounds containing sulfur concerned 2,3,5,6-tetrathiaheptane, dimethyl, trisulfide and disulfide, and methyl [129].

Raw shiitake mushrooms are almost odorless, therefore, mushroom aroma is created in enzymatic and non-enzymatic reactions. Sulfur volatiles involving straight-chain dimethyl disulfide, dimethyl trisulfide, 1-(methylthio)dimethyl disulfide, and cyclic sulfur compounds, including 1,2,4-trithiolane and lenthionine, are formed by enzymatic processes. The formation of cyclic sulfur compounds via non-enzymatic reactions, such as polymerization and degradation, is also possible for example formation 1,2,4-trithiolane from dimethyl disulfide [122].

### 6.2. Blanching

The main aim of blanching is the deactivation of degradation enzymes by short-term heat treatment. Blanching also eliminates air and inhibits oxidative processes, and it can remove some food-spoilage microorganisms. Generally, the temperature is set at a range of 85–100 °C (in water, steam, and less often by microwaving or application of infrared or radio waves). The thermal process takes up 60 to 150 s, and it is followed by rapid cooling, for example, by immersing the product in cold water. This short-term heating affects the composition and sensory properties, for example, blanching Brussels sprouts caused a significant decrease in the dry matter, antioxidant activity, total polyphenol, and ascorbic acid content [131,132]. Among the heat treatment processes, blanching is one of the mildest and recommended to minimize the loss of GLSs and their derivatives [133].

Despite the short treatment period with temperature, the content of GLSs in cabbage after blanching in relation to fresh vegetables decreased [134]. The loss of GLSs may be explained by leaching into the heating water, enzymatic hydrolysis, or thermal degradation of GLSs [135]. The use of blanching for radish also caused a reduction in the number of volatiles compounds, in comparison with raw radish. Concerning organosulfur compounds, the amount of thiophene decreased three times; 1-menthen-8-thiol was observed at a similar level using E-nose analysis. Gas chromatography with mass spectrometry showed that there are no consistent trends in major sulfur volatile compounds between raw and blanched vegetables, with respect to specific classes of compounds. For example, the contents of methyl-, 3-butenyl-, hexyl-, 3-methylthiopropyl- isothiocyanate increased after blanching, whereas pentyl-, 4-methylpentyl- isothiocyanate and erucin, and berteroin had lower values after blanching. However, the content of sulfides during blanching decreases, which is best illustrated by dimethyl disulfide, dimethyl trisulfide, methyl (methylthio)methyl disulfide, and dimethyl tetrasulfide [136]. 

### 6.3. Cooking

Some *Brassica* vegetables are not commonly eaten raw but, after processing, are most often by cooking. Unfortunately, food processing, especially thermal processes, significantly changes the composition of bioactive and odor-active compounds, as well as the taste of the vegetable itself. Increased temperature also affects the possibility of denaturing the myrosinase enzyme and ESP. The inactivation of myrosinase and ESP mainly leads to an increasing trend in the production of nitriles relative to ITCs; however, in general, few hydrolysis products are formed from GLSs. Cooking promotes the formation of sulfur volatiles with the typical sulfurous odor of boiled Brassica vegetables, which is mainly related to the release of sulfides, such as dimethyl sulfide. Unfortunately, the effect of cooking on changing the taste is unknown [41].

Boiling in water is the most common method of cooking shiitake mushrooms. Changes in volatile compounds during boiling in water at 70 °C depend on the cooking time and on the form of the mushrooms used (raw or dried). In general, the short cooking time of raw mushrooms (up to 1 h) favors an increase in the concentration of volatile compounds; only the amount of 1-octen-3-one and dimethyl trisulfide decreased. A similar tendency was noticed during the cooking of dried mushrooms, however, apart from dimethyl trisulfide, the concentration of lenthionine decreased in the first hour of cooking. In the case of using raw mushrooms, long-term cooking (for 3 h) resulted in a decrease in the concentration of aroma-active compounds, such as 1-octen-3-one, 3-octanone, dimethyltrisulfide, 1-(methylthio)dimethyl disulfide, and 1,2,4,5-tetrathiane, while the concentration of dimethyl disulfide, 1-octen-3-ol, and 1,2,4-trithiolane, increased. In contrast, most long-term cooking of dried mushrooms increased in the number of volatile compounds, however, a decrease in the amount of three of them (dimethyl trisulfide, 1,2,4,5-tetrathiane, and lenthionine) was also observed. What is more, lenthionine is unstable during water boiling [122].

### 6.4. Steaming

Steaming involves exposing a vegetable to the steam generated from boiled water, whereby the vegetable is separated from the boiling water with which it is not in direct contact. Steaming is milder than cooking in boiling water; additionally, it resulted in lower rates of cell lysis and myrosinase inactivation. Steaming broccoli, green cabbage, cauliflower, and Brussels sprouts for 20 min has no significant effect on the total GLSs content, unlike cooking. During the first two minutes of the steaming process, myrosinase activity remains unchanged, but as steaming progresses, after 7 min, myrosinase activity is lost by 90.4% [137].

Studies have shown that ESP usually denatures at a lower temperature than myrosinase, which results in the advantage of ITCs over nitriles in volatile compounds obtained as a result of the breakdown of GLSs [41]. The steaming of broccoli and cauliflower decreased the overall odor profile in both vegetables with a reduction in the intensity of most volatiles but sulfur volatiles after steaming were more perceptible. However, the process favors the production of dimethyl trisulfide, which becomes the dominant odorant in steamed vegetables. The odor intensity of ITCs created from corresponding GLSs increased three times, in comparison with raw vegetables, which confirms that the activity of myrosinase during steaming was retained [138].

While the use of steaming is commonly used in the preparation of vegetables, no use of steaming has been found for shiitake mushrooms or truffles.

### 6.5. Frying

Mushrooms need to be processed (e.g., cooked or fried) as food because they are not usually eaten raw. The heat treatment process favors the conversion of non-volatile precursors into volatile compounds. Nine odor active compounds were lost or degraded during frying and one of these compounds was methional. The greatest losses were related to the compounds responsible for the fungal odor of 1-octen-3-one and phenolic from 2-methoxyphenol. On the other hand, the concentration of several sulfur compounds were characteristic of the smell of shiitake mushrooms, e.g., lenthionine, dimethyl trisulfide, and 1,2,4,5-tetrathiane. Moreover, after frying, an increase in the compounds responsible for the desired odor notes was observed, such as caramel-like from 4-hydroxy-2,5-dimethyl-3(2*H*) -furanone and fatty fried from (*E*,*E*) -2,4-decadienal [139].

Vegetables are often prepared by frying by preheating oil in a pan and adding the selected vegetable. Stir-frying *Brassica* vegetables, such as broccolini or kale, reduces the total amount of GLSs. The loss of GLSs in frying is not as great as in cooking, where GLSs leaks directly into the water, which is the heating medium. Interestingly, frying these vegetables does not cause a considerable decrease in the content of ITCs, such as sulforaphane in broccolini or iberin content in kale, which means that the active enzyme myrosinase remained in the vegetable tissues even after the heating treatment was completed. A slight decrease in the number of ITCs compared, for example, to another thermal process, which is cooking, means that frying and steaming are classified as mild heating methods [140].

### 6.6. Freezing

Freezing is one of the most popular food processing methods, mainly used to extend its shelf-life. The quality of the frozen product depends on many factors, including the quality of the product itself, and the speed of the freezing process. Quick freezing leads to large amounts of small ice crystals and less tissue disruption, as opposed to slow freezing, which causes the formation of large intracellular crystals and the destruction of vegetable tissue) [132]. Freezing Brussels sprouts have a major impact on the biochemical pathways and, as a consequence, the volatile profile and aroma properties of raw vegetable samples. After freezing, reduced concentrations were observed for alcohols and bioactive ITC compounds; nevertheless, the number of nitriles, and aldehydes increased considerably. Changes in the ratio of alcohols to aldehydes are related to lipoxygenase activity, however, the increase in the number of nitriles to ITCs after freezing remains unclear at the moment. To maintain the health-promoting properties of Brassica vegetables (e.g., high content of ITCs), it is recommended to consume them when they have not been previously frozen [141].

Freezing has a significant effect on the truffle flavor, as opposed to the negligible effect of storing the mushrooms at the same time, but at a temperature of 4 °C [142]. Descriptive sensory analysis has shown that the aroma of black truffles can be defined by eight major descriptors, such as characteristic fresh truffle aroma, sulfurous, mushroom-like, moldy, animal-like, boiled potatoes-like, buttery, and cheesy. It has been observed that even the mildest freezing conditions at −20 °C for 24 h caused significant changes in the aroma of black truffles. More precisely, the characteristic aroma of truffles was reduced, but the intensity of the aroma remained at the same level due to the increase in odor notes, such as sulfurous, mushroom-like, boiled potatoes-like. The change in the odor of the frozen samples compared to the fresh truffle samples was explained on the basis of quantitative analysis. Frozen samples contained higher amounts of such compounds as diacetyl, 1-octen-3-one, 1-octen-3-ol, 2-methylisoborneol, and dimethyltrisulfide; however, isoamyl alcohol, ethyl 3-methylbutyrate, and methionol decreased [143]. Summing up, freezing has a significant impact on the qualitative (descriptive quantitative analysis) and quantitative impact on the aroma of truffles [7].

### 6.7. Fermentation

Fermentation is both a food preservation method and a food processing method (improving not only the structure but also the flavor) that has been used since ancient times. The microorganisms are capable of processing some of the metabolites present in food by fermentation, resulting in the formation of novel flavor compounds, which impart a unique texture, flavor, and aroma to the processed food. It is mainly bacteria, which produce lactic acid or acetic acid, fungi (mostly yeasts), or molds (species *Mucor*, *Aspergilus*), that are used in food fermentation processes [144].

Brassica fermentation is a very popular food processing method to improve the flavor of raw vegetables. During fermentation, biochemical reactions and transformations occur with the participation of vegetable enzymes (e.g., myrosinase), native bacteria, and added fermentation microorganisms [145]. GLSs concentration decreases during the fermentation process, which is most likely the result of GLSs biotransformation into other metabolites. No GLSs was found in sauerkraut and fermented white cabbage, as GLSs degrades quickly, from 2 to 5 days of fermentation. Fermentation carried out by lactic acid bacteria not only produces lactic acid but also enriches the fermentation product with bioactive compounds. What is more, fermentation supports the biotransformation of glucoraphanin to sulforaphane [146]. A synergistic effect of microorganisms (*Lactobacillus plantarum* and *Leuconostoc mesenteroides*) was also observed, which resulted in a 16-fold increase in sulforaphane concentration in fermented white cabbage, compared to samples where the microorganisms were used separately [147]. 

In order to refine the characteristic mushroom aroma of shiitake mushrooms, the food industry uses various food processing methods, including fermentation. The results of fermented shiitake mushroom samples showed that the acidity, total free amino acid content, and total flavor nucleotides increased throughout the fermentation process, indicating that *Saccharomyces cerevisiae*, *Aspergillus oryzae*, *Aspergillus niger*, and *Lactobacillus plantarum* species had the ability to improve the flavor substances of the shiitake mushroom. *Lactobacillus plantarum* species showed the greatest influence on the improvement of taste and aroma in the fermentation process. The taste activity value of free amino acids and flavor nucleotides were both higher than one, suggesting that those flavor substances have a significant influence on the fermented food taste [144].

## 7. Conclusions and Perspective

Over the years, there have been many attempts to determine what molecular determinants affect the smell of chemical compounds—molecular mass, polarity, presence of hydrogen bonding, stability, symmetry of the molecule, and volatility. Sulfur as a heteroatom in molecules, compared to an oxygen atom from the same group of the periodic table, significantly increases sensory properties. It is believed that this effect is associated with the electronic capabilities of these two atoms. Unlike oxygen, sulfur has the option of expanding d-orbital to 10 electrons on the valence shell [11].

Flavor research indicates that VSCs are responsible for the remarkable flavor of many food products. For vegetables of Allium and Brassica, but also for some mushroom they are crucial for their characteristic aroma. However, considering the dynamic formation of sulfur-containing volatiles in enzymatic reactions, their instability, and rapid decomposition of some intermediates, analysis of them is extremely challenging. It starts from matrix issues, easily undergoing oxidation processes, low detection limits required to quantify aroma relevant concentrations, and also the possibilities of the creation of artefacts in gas chromatography injection port. VSCs ale listed among the most potent odorants found in raw materials and foods. With their extremely low odor thresholds, they contribute to the characteristic flavor of many foods. However, their very often low odor thresholds and low concentrations make the analytical process difficult. For vegetables, especially Brassica, further research using the sensomics approach is necessary to fully explore their flavor potential. There is still insufficient data on odor/taste thresholds of many VSC and even GLSs to fully explore the flavor of Brassica.

## Figures and Tables

**Table 1:** Selected sulfur key odorants in food formed in enzymatic and chemical (mainly thermal) reactions.

Name	Structure	Odor Threshold	Odor Quality	Occurence	Name	Structure	Odor Threshold	Odor Quality	Occurence
diallyl thiosulfinate (allicin)	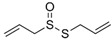	NA	Freshly crushed garlic	Garlic, onion	3-(methylthio)propanal (methional)	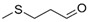	1.3 µg/L in water	Cooked potato-like	Beef, truffle, coffee, cheese
benzenemethanethiol(phenylmethanethiol)	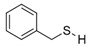	0.01 µg/L in 67% ethanol-water solution	Roasted, garden cress seed	Beef, wine, Baijiu	2-furfurylthiol(2-furanmethanethiol)	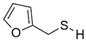	0.004 µg/kg in water-alcohol solution	Sulfuric, burnt	Popcorn, coffee, meat, sesame seeds
1,2,4-trithiolane	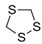	1.27 µg/L in water	Shiitake-like, sulfury, onion-like	Shiitake mushrooms, truffle, stinky beans	bis-(2-methyl-3-furyl)disulfide(1,2-bis(2-methyl-3-furanyl)disulfane)	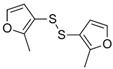	0.00002 µg/L in water	Meat-like	Beef, coffee, brown rice, tea, kohlrabi
4-mercapto-4-methyl-2-pentanone	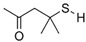	0.0001 µg/L in water	Blackcurrant-like, sulfury	Green tea, edible flowers	dimethyl trisulfide (DMS)	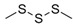	0.009 µg/L in water	Sulfuric, cabbage-like	Brassica vegetables, wine, cheese, coffee
1-p-menthene-8-thiol	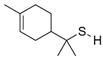	0.0002 µg/L in water	Grapefruit-like, sulfuric	Grapefruit, orange, honey pomelo	methanethiol	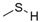	0.59 µg/L in water	Sulfuric, cabbage-like	Coffee, cheese, potato, spinach, durian
2-acetyl-2-thiazoline	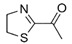	1 µg/L in water	Roasted, popcorn-like	Meat, rice, shiitake mushroom	3-methyl-2-butene-1-thiol	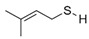	0.00076 µg/L in water	Beer-like, animal-like	Beer, cofee, durian

NA—not available. Based on references [18,19,20,21,22,23,24,25,26].

## Data Availability

Not applicable.

## Supplementary Materials

The following supporting information can be downloaded at https://www.mdpi.com/article/10.3390/molecules27186116/s1. Table S1. Variety of Sulfur Compounds in Vegetables and Mushrooms.
