# Supplementary material for: Role of Sulfur Compounds in Vegetable and Mushroom Aroma
Source: Molecules. 2022 Sep 19;27(18):6116. doi: 10.3390/molecules27186116 (PMC9502545; doi:10.3390/molecules27186116)
Supplement: Supplementary file 1 [file molecules-27-06116-s001.zip › molecules-1848119-supplementary.pdf]

Role of sulfur compounds in vegetable and mushroom aroma

Monika A. Marcinkowska, and Henryk H. Jeleń,*

Faculty of Food Science and Nutrition, Poznań University of Life Sciences, Wojska Polskiego 31, 60-624 Poznań, Poland; monika.marcinkowska@up.poznan.pl
* Correspondence: henrykj@up.poznan.pl; Tel.: +48-618-487-273

Supplementary file

Table S1. Variety of sulfur compounds in vegetables and mushrooms.

Class of Sulfur Compound	Name	Occurrence in vegetable (family)	Odor quality	Reference
Isothiocyanates	allyl isothiocyanate	*Brassicaceae*	pungent, onion-like	[1,2]
	3-butenyl isothiocyanate	*Brassicaceae*	pungent, garlic-like	[1,2]
	benzyl isothiocyanate	*Brassicaceae*	herb-like, sweaty, pungent	[1,2]
	1-isothiocyanato-4-(methylsulfanyl)butane	*Brassicaceae*	mushroom-like, broth-like	[1]
	(2-isothiocyanatoethyl)benzene	*Brassicaceae*	broth-like, vegetable-like	[1]
	isopropyl isothiocyanate	*Brassicaceae*	pungent, grassy	[3]
	sec-butyl isothiocyanate	*Brassicaceae*	radish-like, vegetative	[3]
	isobutyl isothiocyanate	*Brassicaceae*	cooked, pungent, sulphury	[3]
	butyl isothiocyanate	*Brassicaceae*	peppery, sulphurous, oniony	[3]
	isoamyl isothiocyanate	*Brassicaceae*	pungent, grassy	[3]
	4-pentenyl isothiocyanate	*Brassicaceae*	pungent, peppery, sulphurous, musty	[3]
	pentyl isothiocyanate	*Brassicaceae*	cabbage, green, rotten	[3]
	octyl isothiocyanate	*Brassicaceae*	green, vegetative	[3]
Nitriles and Epithionitriles	3-hydroxy-4-pentanenitrile	*Brassicaceae*	nf	[4]
	β-hydroxy-thiiranepropanenitrile	*Brassicaceae*	nf	[4]
	3-butenenitrile	*Brassicaceae*	grassy, pungent	[4]
	2-butenenitrile	*Brassicaceae*	pungent	[5]

	thiiraneacetonitrile	*Brassicaceae*	sweaty, onion-like, pungent	[2–4]
	4-pentanenitrile	*Brassicaceae*	nf	[4]
	3-pentanenitrile	*Brassicaceae*	pungent	[5]
	thiiranepropanenitrile	*Brassicaceae*	nf	[4]
	5-hexanenitrile	*Brassicaceae*	pungent	[4,5]
	thiiranebutanenitrile	*Brassicaceae*	nf	[4]
	phenyl-3-propanenitrile	*Brassicaceae*	nasturtium	[4,6]
	3-methyl-3-butenenitrile	*Brassicaceae*	nf	[5]
	5-(methanesulfinyl)pentanenitrile	*Brassicaceae*	broth-like, onion-like, sulfurous	[1]
	4-(methylsulfanyl)butanenitrile	*Brassicaceae*	broth-like, garlic-like, sulfurous	[1]
	5-(methylsulfanyl)pentanenitrile	*Brassicaceae*	broccoli-like, cabbage-like	[1]
	phenylacetonitrile	*Brassicaceae*	mushroom-like	[1]
	5-methylhexanenitrile	*Brassicaceae*	nf	[3]
	6-heptenenitrile	*Brassicaceae*	nf	[3]
	benzenepropanenitrile	*Brassicaceae*	herbal, green, floral	[3]
Sulfides and Polysulfides	hydrogen sulfide	*Brassicaceae, Caprifoliaceae, Amaranthaceae*	rotten eggs	[7,8]
	dimethyl sulfide	*Brassicaceae, Amaryllidaceae, Caprifoliaceae, Amaranthaceae, Asteraceae, Solanaceae, Tuberaceae*	cooked asparagus-like, putrid	[7–13]
	dimethyl disulfide	*Brassicaceae, Amaryllidaceae, Solanaceae, Tuberaceae*	cabbage-like, sulfuric	[1,7,11–14]
	dimethyl trisulfide	*Brassicaceae, Amaryllidaceae, Fabaceae, Solanaceae, Tuberaceae*	sulfuric, cabbage-like	[1,7,10,12–14]
	dimethyl tetrasulfide	*Amaranthaceae*	cabbage-like, rotten	[7,15]
	methyl 2-methyl-3-furyl disulfide	*Brassicaceae*	meat-like, cabbage-like	[1]
	methyl propyl disulfide	*Amaryllidaceae*	onion-like	[7,16]
	propyl allyl disulfide	*Amaryllidaceae*	onion-like	[16,17]
	dipropyl disulfide	*Amaryllidaceae*	onion-like	[7,16]
	methyl propyl trisulfide	*Amaryllidaceae*	onion-like, cabbage-like	[7,16]
	dipropyl trisulfide	*Amaryllidaceae*	onion-like, metallic	[7,16]

	2-propenyl propyl trisulfide	*Amaryllidaceae*	onion-like	[16,17]
	ethyl methyl disulfide	*Amaryllidaceae, Solanaceae*	white truffles-like	[11,14,18]
	diallyl sulfide	*Amaryllidaceae*	garlic-like	[7,14]
	allyl methyl disulfide	*Amaryllidaceae*	garlic-like	[7,14]
	ethyl propyl disulfide	*Amaryllidaceae*	sulphurous, green, vegetable-like	[6,14]
	butyl methyl disulfide	*Amaryllidaceae*	nf	[14]
	ethyl methyl trisulfide	*Amaryllidaceae*	garlic-like, green, onion-like	[6,14]
	diallyl disulfide	*Amaryllidaceae*	garlic-like	[7,14]
	dipropyl disulfide	*Amaryllidaceae*	onion-like	[7,14]
	allyl methyl trisulfide	*Amaryllidaceae*	garlic-like	[7,14]
	methyl propyl trisulfide	*Amaryllidaceae*	onion-like, cabbage-like	[7,14]
	dimethyl tetrasulfide	*Amaryllidaceae*	sulfurous	[6,14]
	butyl methyl trisulfide	*Amaryllidaceae*	nf	[14]
	diallyl trisulfide	*Amaryllidaceae*	garlic-like	[7,14]
	dipropyl trisulfide	*Amaryllidaceae*	onion-like, metallic	[7,14]
	methyl pentyl tetrasulfide	*Amaryllidaceae*	nf	[14]
	dipropyl tetrasulfide	*Amaryllidaceae*	cooked onion-like	[7,14]
	allyl propyl tetrasulfide	*Amaryllidaceae*	nf	[14]
	propyl methyl pentasulfide	*Amaryllidaceae*	nf	[14]
	bis(methylthio)methane	*Tuberaceae*	white truffle-like	[12,13]
Thiols	pentane-1-thiol	*Brassicaceae*	burned, rubber-like	[1]
	(1S)-1-phenylethane-1-thiol	*Brassicaceae*	burned	[1]
	2-phenylethane-1-thiol	*Brassicaceae*	rubber-like	[1]
	1-propanethiol	*Amaryllidaceae*	leek-like, onion-like	[17]
	2-propene-1-thiol	*Amaryllidaceae*	garlic-like	[10]
	methanethiol	*Brassicaceae, Caprifoliaceae, Amaranthaceae, Solanaceae, Tuberaceae*	sulfurous, gasoline-like, garlic-like	[8,11,13,19]
	ethanethiol	*Solanaceae*	rotten, onion-like	[7,11]
	butanethiol	*Solanaceae*	garlic-like, burned, rubber-like	[7,11]
	1-menthen-8-thiol	*Brassicaceae*	grape-like, resinous, woody	[20]
Miscellaneous	methionol	*Brassicaceae, Liliaceae, Tuberaceae*	cooked potato-like	[1,21,22]
	methional	*Brassicaceae, Amaryllidaceae,*	cooked potato-like	[7,12,13,23–25]

	Solanaceae, Tuberaceae		
thiophene-2-carbaldehyde	*Brassicaceae*	roasty	[1]
4-methyl-5-thiazoleethanol	*Brassicaceae*	broth-like, sulfurous, nutty	[1]
dimethyl sulfone	*Brassicaceae*	sulfurous	[19]
dimethyl sulfoxide	*Brassicaceae, Asteraceae, Tuberaceae*	garlic-like, mushroom-like	[9,19,22]
1,3-thiazole	*Brassicaceae*	roasty	[5]
5-methyl-1,3-thiazole	*Brassicaceae*	roasty, meat-like	[5]
5-ethyl-1,3-thiazole	*Brassicaceae*	roasty	[5]
2-methyl-3-furanthiol	*Brassicaceae, Tuberaceae*	meat-like, onion-like	[5,12]
S-methyl thioacetate	*Liliaceae*	warm, cooked-like	[21]
2,4-dimethylthiophene	*Amaryllidaceae*	nf	[16]
2,5-dimethylthiophene	*Amaryllidaceae*	nutty, sulfurous	[6,16]
3,4- or 2,4-dimethyl thiophen	*Amaryllidaceae*	wood, dry smell, green, bookstore	[17]
2-acetylthiazole	*Brassicaceae, Solanaceae*	roasty, popcorn-like	[1,7,11]
benzothiazole	*Brassicaceae, Asteraceae, Solanaceae*	rubber-like, cabbage-like	[1,9,11]
cycloalliin	*Amaryllidaceae*	nf	[26]
S-methyl-L-cysteine sulfoxide (methiin)	*Brassicaceae, Amaryllidaceae*	odourless	[26–28]
S-propyl-L-cysteine sulfoxide (propiin)	*Amaryllidaceae*	odourless	[26,27]
S-allyl-L-cysteine sulfoxides (alliine)	*Amaryllidaceae*	odourless	[26,27]
S-propenyl-1-L-cysteine sulfoxides (isoalliin)	*Amaryllidaceae*	odourless	[26,27]
2-propenyl sulfenic acid	*Amaryllidaceae*	nf	[29]
(*E*)-1-propenyl sulfenic acid	*Amaryllidaceae*	nf	[29]
diallyl thiosulfinate (allicin)	*Amaryllidaceae*	freshly crushed garlic-like	[30]
methylmethanethiosulfinate	*Brassicaceae, Amaryllidaceae*	freshly cut onion-like	[27,31]
1-propenyl thiosulfinates	*Amaryllidaceae*	freshly cut onion-like	[27,31]
3-vinyl-4*H*-1,2-dithiin	*Amaryllidaceae*	garlic-like	[23]
2-vinyl-4*H*-1,3-dithiin	*Amaryllidaceae*	pungent, garlic-like	[23]
5-methyl-2-thiophenecarboxaldehyde	*Amaryllidaceae*	aniseed-like	[23]
1,2,4-trithiolane	*Omphalotaceae*	sulfury, onion-like	[7,32]
1,2,4,5-tetrathiane	*Omphalotaceae*	sulfury, burned	[7,32]

lenthionine	*Omphalotaceae*	sulfury, burned	[32,33]
hexathiepane	*Omphalotaceae*	nf	[32]
cyclic octaatomiac sulfur	*Omphalotaceae*	nf	[32]
2-methyl-4,5-dihydrothiophene	*Tuberaceae*	aged cheese-like, rubber-like	[12,13]
3-methyl-4,5-dihydrothiophene	*Tuberaceae*	onion-like, truffle-like	[12]

nf, not found.

1. Marcinkowska, M.; Frank, S.; Steinhaus, M.; Jeleń, H.H. Key Odorants of Raw and Cooked Green Kohlrabi (Brassica oleracea var. gongylodes L.). *J. Agric. Food Chem.* **2021**, *69*, 12270–12277, doi:10.1021/acs.jafc.1c04339.

2. Kroener, E.M.; Buettner, A. Sensory-analytical comparison of the aroma of different horseradish varieties (Armoracia rusticana). *Front. Chem.* **2018**, *6*, 1–11, doi:10.3389/fchem.2018.00149.

3. Bell, L.; Kitsopanou, E.; Oloyede, O.O.; Lignou, S. Important odorants of four brassicaceae species, and discrepancies between glucosinolate profiles and observed hydrolysis products. *Foods* **2021**, *10*, doi:10.3390/foods10051055.

4. Collett, M.G.; Stegelmeier, B.L.; Tapper, B.A. Could nitrile derivatives of turnip (Brassica rapa) glucosinolates be hepato- or cholangiotoxic in cattle? *J. Agric. Food Chem.* **2014**, *62*, 7370–7375, doi:10.1021/jf500526u.

5. Jia, X.; Wang, L.; Zheng, C.; Yang, Y.; Wang, X.; Hui, J.; Zhou, Q. Key Odorant Differences in Fragrant Brassica napus and Brassica juncea Oils Revealed by Gas Chromatography–Olfactometry, Odor Activity Values, and Aroma Recombination. *J. Agric. Food Chem.* **2020**, *68*, 14950–14960, doi:10.1021/acs.jafc.0c05944.

6. Nursten, H.E. *The Maillard Reaction*; The Royal Society of Chemistry, 2005; ISBN 978-0-85404-964-6.

7. Kreissl, J; Mall, V; Steinhaus, P; Steinhaus, M. Leibniz-LSB@TUM Odorant Database, Version 1.0. Leibniz-Institute for Food Systems Biology at the Technical University of Munich: Freising, Germany Available online: https://www.leibniz-lsb.de/en/databases/leibniz-lsbtum-odorant-database (accessed on May 18, 2021).

8. Dryahina, K.; Som, S.; Smith, D.; Španěl, P. Characterization of spoilage-related volatile organic compounds in packaged leaf salads. *Flavour Fragr. J.* **2020**, *35*, 24–33, doi:10.1002/ffj.3535.

9. Deza-Durand, K.M.; Petersen, M.A. Volatile compounds of modified atmosphere packaged cut iceberg lettuce: Effect of extremely low O2, season, cultivar and storage time. *Food Res. Int.* **2014**, *62*, 254–261, doi:10.1016/j.foodres.2014.02.017.

10. Van Ruth, S.M.; Roozen, J.P.; Cozijnsen, J.L.; Posthumus, M.A. Volatile compounds of rehydrated French beans, bell peppers and leeks. Part II. Gas chromatography/sniffing port analysis and sensory evaluation. *Food Chem.* **1995**, *54*, 1–7, doi:10.1016/0308-8146(95)92655-4.

11. Dresow, J.F.; Böhm, H. The influence of volatile compounds of the flavour of raw, boiled and baked potatoes: Impact of agricultural measures on the volatile components. *Landbauforsch. Volkenrode* **2009**, *59*, 309–337.

12. Vahdatzadeh, M.; Deveau, A.; Splivallo, R. The role of the microbiome of truffles in aroma formation: A meta-analysis approach. *Appl. Environ. Microbiol.* **2015**, *81*, 6946–6952, doi:10.1128/AEM.01098-15.

13. Splivallo, R.; Ottonello, S.; Mello, A.; Karlovsky, P. Truffle volatiles: From chemical ecology to aroma biosynthesis. *New Phytol.* **2011**, *189*, 688–699, doi:10.1111/j.1469-8137.2010.03523.x.

14. Pino, J.A.; Fuentes, V.; Correa, M.T. Volatile constituents of Chinese chive (Allium tuberosum Rottl. ex Sprengel) and rakkyo (Allium chinense G. Don). *J. Agric. Food Chem.* **2001**, *49*, 1328–1330, doi:10.1021/jf9907034.

15. Masanetz, C.; Guth, H.; Grosch, W. Fishy and hay-like off-flavours of dry spinach. *Eur. Food Res. Technol.* **1998**, *206*, 108–113.

16. Chung, M.S. Volatile compounds of the cultivated dumebuchu (Allium senescens L. var. senescens). *Food Sci. Biotechnol.* **2010**, *19*, 1679–1682, doi:10.1007/s10068-010-0238-0.

17. Nielsen, G.S.; Poll, L. Determination of Odor Active Aroma Compounds in Freshly Cut Leek (Allium ampeloprasum Var. Bulga) and in Long-Term Stored Frozen Unblanched and Blanched Leek Slices by Gas Chromatography Olfactometry Analysis. *J. Agric. Food Chem.* **2004**, *52*, 1642–1646, doi:10.1021/jf030682k.

18. Chemical Book. Available online: https://www.chemicalbook.com/ (accessed on 18th January 2022).

19. Zhou, Q.; Tang, H.; Jia, X.; Zheng, C.; Huang, F.; Zhang, M. Distribution of glucosinolate and pungent odors in rapeseed oils from raw and microwaved seeds. *Int. J. Food Prop.* **2018**, *21*, 2296–2308, doi:10.1080/10942912.2018.1514632.

20. Hong, S.J.; Boo, C.G.; Lee, J.; Hur, S.W.; Jo, S.M.; Jeong, H.; Yoon, S.; Lee, Y.; Park, S.S.; Shin, E.C. Chemosensory approach supported-analysis of wintering radishes produced in Jeju island by different processing methods. *Food Sci. Biotechnol.* **2021**, *30*, 1033–1049, doi:10.1007/s10068-021-00948-2.

21. Ulrich, D.; Hoberg, E.; Bittner, T.; Engewald, W.; Meilchen, K. Contribution of volatile compounds to the flavor of cooked asparagus. *Eur. Food Res. Technol.* **2001**, *213*, 200–204, doi:10.1007/s002170100349.

22. Mustafa, A.M.; Angeloni, S.; Nzekoue, F.K.; Abouelenein, D.; Sagratini, G.; Caprioli, G.; Torregiani, E. An overview on truffle aroma and main volatile compounds. *Molecules* **2020**, *25*, 1–27, doi:10.3390/molecules25245948.

23. Abe, K.; Hori, Y.; Myoda, T. Characterization of key aroma compounds in aged garlic extract. *Food Chem.* **2020**, *312*, 126081, doi:10.1016/j.foodchem.2019.126081.

24. Jia, X.; Wang, L.; Zheng, C.; Yang, Y.; Wang, X.; Hui, J.; Zhou, Q. Key Odorant Differences in Fragrant Brassica napus and Brassica juncea Oils Revealed by Gas Chromatography-Olfactometry, Odor Activity Values, and Aroma Recombination. *J.*

Agric. Food Chem. **2020**, *68*, 14950–14960, doi:10.1021/acs.jafc.0c05944.

25. Bough, R.A.; Holm, D.G.; Jayanty, S.S. Evaluation of Cooked Flavor for Fifteen Potato Genotypes and the Correlation of Sensory Analysis to Instrumental Methods. *Am. J. Potato Res.* **2020**, *97*, 63–77, doi:10.1007/s12230-019-09757-0.
26. Goncharov, N.; Orekhov, A.N.; Voitenko, N.; Ukolov, A.; Jenkins, R.; Avdonin, P. *Organosulfur Compounds as Nutraceuticals*; Elsevier Inc., 2016; ISBN 9780128021477.
27. Olech, Z.; Zaborska, W.; Kot, M. Jack bean urease inhibition by crude juices of Allium and Brassica plants. Determination of thiosulfinates. *Food Chem.* **2014**, *145*, 154–160, doi:10.1016/j.foodchem.2013.08.044.
28. Rose, P.; Moore, P.K.; Whiteman, M.; Zhu, Y.Z. An appraisal of developments in Allium sulfur chemistry: Expanding the pharmacopeia of garlic. *Molecules* **2019**, *24*, 1–17, doi:10.3390/molecules24214006.
29. Yoshimoto, N.; Saito, K. S-Alk(en)ylcysteine sulfoxides in the genus Allium: Proposed biosynthesis, chemical conversion, and bioactivities. *J. Exp. Bot.* **2019**, *70*, 4123–4137, doi:10.1093/jxb/erz243.
30. Borlinghaus, J.; Foerster, J.; Kappler, U.; Antelmann, H.; Noll, U.; Gruhlke, M.C.H.; Slusarenko, A.J. Allicin, the odor of freshly crushed garlic: A review of recent progress in understanding allicin's effects on cells§. *Molecules* **2021**, *26*, doi:10.3390/molecules26061505.
31. Løkke, M.M.; Edelenbos, M.; Larsen, E.; Feilberg, A. Investigation of volatiles emitted from freshly cut onions (Allium cepa L.) by real time proton-transfer reaction-mass spectrometry (PTR-MS). *Sensors (Switzerland)* **2012**, *12*, 16060–16076, doi:10.3390/s121216060.
32. Li, S.; Wang, A.; Liu, L.; Tian, G.; Wei, S.; Xu, F. Evaluation of nutritional values of shiitake mushroom (Lentinus edodes) stipes. *J. Food Meas. Charact.* **2018**, *12*, 2012–2019, doi:10.1007/s11694-018-9816-2.
33. Wang, S.L.; Lin, S.Y.; Du, H.T.; Qin, L.; Lei, L.M.; Chen, D. An insight by molecular sensory science approaches to contributions and variations of the key odorants in shiitake mushrooms. *Foods* **2021**, *10*, doi:10.3390/foods10030622.
